# Collecting at-Home Biometric Measures for Longitudinal Research From the i3C: Feasibility and Acceptability Study

**DOI:** 10.2196/71103

**Published:** 2025-06-18

**Authors:** Marta Russell, Erin Cain, Lydia Bazzano, Ileana De Anda, Jessica G Woo, Elaine M Urbina

**Affiliations:** 1 The Heart Institute, Cincinnati Children’s Hospital Medical Center, Cincinnati, OH, United States; 2 Department of Pediatrics, University of Cincinnati College of Medicine, 3333 Burnet Ave, MLC 7002, Cincinnati, OH, 45220, United States, 1 513-636-8265; 3 Department of Epidemiology, School of Public Health, Tulane University, New Orleans, LA, United States; 4 Division of Biostatistics and Epidemiology, Cincinnati Children’s Hospital Medical Center, Cincinnati, OH, United States

**Keywords:** mHealth, cardiovascular risk factors, epidemiology, wearable devices, feasibility, acceptability, biometric

## Abstract

**Background:**

The use of individual wearable devices or internet-based applications to collect biometric data from research participants is popular, but several devices may be needed to replace a full set of research measurements.

**Objective:**

In this study, we assessed the feasibility of a “Virtual Home Clinic” within the context of long-term epidemiologic studies.

**Methods:**

Participants from 3 study cohorts were recruited. Devices were sent to the home to measure anthropometrics, resting metabolic rate, blood pressure (BP), heart rate (HR), heart rhythm, oxygen saturation, glucose, total cholesterol, physical activity, diet, sleep duration or quality, and arterial stiffness over the course of 1 week. Stool and saliva were also self-collected for microbiome, DNA, and cotinine. Feasibility and acceptability of collecting measurements using home devices were assessed.

**Results:**

A total of 134 participants were enrolled (87% female, 31% Black; mean age 54.2, SD 8.4 years). Furthermore, 91% (N=122) performed at least one of the home tests. At least two-thirds of participants were able to complete all of the requested readings for glucose, electrocardiogram, BP, diet record, and resting metabolic rate. The scale that measured weight, body composition, and pulse wave velocity (PWV) was more difficult to use (113/134, 84% participants recorded at least one weight and 84/134, 63% recorded a PWV). The device to measure total cholesterol was least successful (32/134, 24% participants completed all readings, 72/134, 54% provided at least one result). Return of biospecimens was highly successful (115/134, 86% returned saliva and 113/134, 84% returned stool). Of 95 who responded to the user acceptability survey, 38 (40%) participants preferred home assessment, 36 (38%) preferred clinic, and 21 (22%) did not have a preference. The mean user acceptability score across devices for ease of use was 4.3 (SD 1.0), for instructions was 4.5 (SD 0.7), and for time to use was 3.9 (SD 1.1; scale of 1‐5, with higher scores indicating greater acceptability). The study team documented several regulatory or IT, connectivity or account, data retrieval, and logistical issues encountered during the study.

**Conclusions:**

Despite several complications involved with managing multiple devices and applications, most of the components of the virtual home clinic were reasonably feasible and acceptable to participants.

## Introduction

Early life factors play an important role in successful aging [1,2]. However, prospectively collected data is needed to construct accurate trajectories of health status (eg, BMI, blood pressure, and cholesterol levels) and lifestyle factors (eg, diet, physical activity, alcohol, and tobacco use) across the lifespan to determine their association with cognitive and physical function later in life. Many barriers exist in collecting longitudinal data across the lifespan [3]. Importantly, as adolescents age into adulthood, many move away from their initial recruitment site which may reduce generalizability of findings of the remaining cohort from adult follow-up visits. Mobile health (mHealth) approaches have been proposed as a means to overcome some of these barriers [4], but the feasibility of collecting a broad range of measurements using at-home devices has not been sufficiently evaluated in this setting. Therefore, we conducted a study to evaluate the feasibility of a “Virtual Home Clinic” by sending biometric measurement devices to participants’ homes. We also sought to determine the acceptability of the virtual home clinic approach compared with a research clinic visit. Separately, we will seek to correlate measures obtained in the virtual home clinic to those in the research clinic. Our goal is to establish the feasibility and acceptability of using a suite of wearable technology and online applications to conduct “remote” study visits in epidemiologic studies to improve the sustainability of longitudinal studies whose study population has dispersed over time.

## Methods

### Population

Three cohorts that are members of the International Childhood Cardiovascular Cohort Consortium (i3C) [5] participated in this study: the Bogalusa Heart Study (BHS), the National Heart, Lung, and Blood Institute (NHLBI) Growth and Health Study (NGHS), and the Princeton Lipid Research Study (PLRS). The most recent phone follow-up of these cohorts this project was completed in 2019 [6].

The BHS which began in 1973 conducted 9 cross-sectional cardiovascular risk factor screenings of children aged 3 to 18 years between 1973 and 1992 in a community of Black and White men and women of Bogalusa, LA, with participation rates of 80% to 93%. In addition, 10 cross-sectional screenings of young adults aged 19‐52 years who had previously been examined as children have been conducted to date, with participation rates of 72% to 93%. Cardiovascular risk factors (adiposity, BP, and cholesterol levels) and noninvasive measures of cardiac and vascular health (carotid intima media thickness and arterial stiffness) [7].

The NGHS was initiated in 1987 as a study of racial (Black-White) differences in the development of cardiovascular risk factors in girls in Cincinnati, Ohio [7]. All participants were between the ages of 9 and 10 years at baseline and followed annually or biannually to age 28 years. Cardiovascular risk factors, echocardiography and dual-energy x-ray absorptiometry (DXA) and abdominal magnetic resonance imaging (MRI) for adiposity were collected.

The PLRS was initiated in 1973 as part of the NHLBI Lipid Research Clinics Study [8]. The study enrolled children in first to 12th grades in the Princeton City School District of Cincinnati, Ohio, plus a 50% random sample of the students’ parents (by household). Three study visits were conducted between 1973 and 1978 to collect a variety of cardiovascular risk factors. Between 1998 and 2003, 911 former school children participated in another in-person visit at mean age 36.

For the current feasibility study of the virtual home clinic, 50 participants from each cohort living near the study sites, with access to Wi-Fi at home, and who were willing to attend an in-person visit at the study sites were invited to participate. Procedures were explained, and informed consent was obtained electronically. These 50 per cohort were invited from a subset of 150 per cohort who had previously enrolled in a pilot study testing the feasibility of phone administration of neurocognitive evaluations. Enrollment into this virtual home clinic study thus represents a convenience sample of contactable and willing participants. The study protocol was approved by the institutional review boards of the participating institutions.

### Data Collection

Participants were asked to complete the virtual home clinic and an in-person research clinic visit for the validation portion of the study. The virtual home clinic study kit was either mailed to a participant (if they conducted the virtual home clinic C first) or given to the participant at the end of their in-person visit. The virtual home clinic kit included detailed instructions, staff contact information, an iPad (Apple Inc) with device apps and instructional videos preloaded, a color-coded schedule of activities, and shipping materials for returning the kit. The virtual home clinic study protocol included 6 devices, 1 iPad app and 3 sample collections (Table 1).

**Table 1.** Home devices and online applications included in the virtual home clinic.

Mobile technology devices and measurement domains and variables		Data retrieval method
Withings Body Cardio Scale		Log into participant’s account in a browser and request data download. Click link in email to download a zip folder containing data in three separate CSV^a^ files.
	Weight	
	Body composition: %body fat, fat mass, lean mass	
	Arterial stiffness: PWV^b^	
Breezing Pro device		
	Resting metabolic rate: resting energy expenditure (measured and predicted)	Log into participant’s account in app. Select option to export entire history. Follow link in email to download CSV file.
AliveCor KardiaMobile 6L		
	6-lead ECG^c^: heart rate and rhythm (normal, sinus, bradycardia, and atrial fibrillation)	Log into participant’s account in app. Download PDF of each ECG completed, then export each PDF via email. Save PDFs and enter results in study database.
QardioArm Home BP^d^ Monitor		
	Blood pressure; systolic and diastolic blood pressure and heart rate	Log into participant’s account in app. Choose the “Send History” option and enter an email address. CSV file of results will be emailed to that address.
AimStrip Tandem		
	Laboratory assays: glucose and total cholesterol	Use buttons on meter to view each test result. Enter results in study database.
GB HealthWatch 360		
	Dietary intake: mean total calories per day, % calories from fat, total sodium per day, and total added sugars per day	Log into study’s HealthWatch portal in browser. Generate and download report for relevant dates. Data exported in two CSV files in a zip folder.
Garmin Vivosmart 4		
	Physical activity: number of steps, minutes of intense movement, and heart rate	Log into participant’s account in browser. Under “Reports,” generate and download CSV files for each measure. Date formats may need to be adjusted.
	Sleep: time in REM^e^ + deep sleep and oxygen saturation	In browser, view sleep and oxygen saturation data and save a screenshot for each night. Enter results in study database.
DNA Genotek OMNIgene GUT OMR-200 stool collection		
	Microbiome (16s): % collected	Sample collection status recorded in spreadsheet.
Abbott Quantisal saliva collection		
	Cotinine	Samples sent to LabCorp for processing. Retrieve results from study’s LabCorp portal and enter results in study database.
DNA Genotek Oragene OG-500 saliva collection		
	DNA: % collected	Sample collection status recorded in spreadsheet.

a CSV: comma separated values.

b PWV: pulse wave velocity.

c ECG: electrocardiogram.

d BP: blood pressure.

e REM: rapid eye movement

Together, the devices were selected to measure key cardiovascular parameters that would be included in a standard in-person follow-up visit. Requested measurements included 3 glucose and total cholesterol readings with the AimStrip Tandem (AimStrip), 3 electrocardiogram tracings with the Alivecor KardiaMobile 6L (Kardia), 7 weight or body composition and 3 pulse wave velocity (PWV) readings with the Withings Body Cardio scale (Withings), and 3 resting metabolic rate measurements with the Breezing Pro device (Breezing; Cincinnati cohorts only). One week of daily diet tracking was requested using the GB HealthWatch 360 app (HealthWatch 360), and participants were asked to wear a Garmin Vivosmart 4 fitness tracker watch (Garmin) for the entire week to measure activity (steps, minutes with elevated heart rate), oxygen saturation during sleep, and sleep duration and quality. In addition, 3 sets of 3 BP and HR measurements (total of 9) were requested using the QardioArm Home BP monitor (Qardio). Three biologic samples were requested: Abbott Quantisal saliva collection (Quantisal), DNA Genotek Oragene OG-500 saliva collection (Oragene) and DNA Genotek OMNIgene GUT OMR-200 stool collection (OMNIgene). Subjects were asked to return the kit and samples after 1 week. Once device data was retrieved, a full reset was performed on all hardware to ensure privacy between participants. For additional security, new iPad app accounts were created for each participant using only their unique study identifier and the demographics required by the app (eg date of birth, sex, height, and weight). Reset procedures were rigorously tested to confirm effective deletion of all data, and were documented in detail in study manuals. All kit components were thoroughly cleaned using hospital protocols before and after each virtual home clinic.

A questionnaire to assess acceptability, ease of use, and functionality of each device was sent to all participants electronically after completion of the virtual home clinic. User acceptability was assessed with the questions “Was this device or app easy to use?” (1=Very difficult to use; 2=Somewhat difficult; 3=Neither difficult nor easy; 4=Somewhat easy; and 5=Very easy); “Were the instructions easy to follow?” (same scoring); “How do you feel about the time it took to use the device or app?” (1=Took much longer than I would have liked; 2=Took somewhat longer; 3=Took about as long as I expected; 4=Took somewhat less time than I expected; and 5=Took very little time).

### Statistical Analyses

Feasibility was assessed as proportion of requested in-home tests completed, and percent of requested saliva and stool samples returned. Mean scores by device and overall were calculated. Due to small sample size, differences by cohort, sex and race were not assessed.

### Ethical Considerations

Each center obtained approval from their local institutional review board (IRB) and all participants signed an informed consent. All data was identified with only a participant ID number (deidentified). Participants were compensated $200 for completion of all arms of the study.

## Results

### Population

A total of 134 participants consented to participate (Table 2).

**Table 2.** Description of the study population. Three participants did not have laboratory evaluation.

BHS^a^ (n=41)	NGHS^b^ (n=52)	PLRS^c^ (n=41)	All (n=134)
Age (years), mean (SD)	57.1 (6.7)	45.5 (0.6)	62.3 (3.4)	54.2 (8.4)
Race (White), n (%)	34 (82.9)	25 (48.1)	34 (82.9)	93 (69.4)
Sex (male), n (%)	7 (17.1)	0 (0)	11 (26.8)	18 (13.4)
In-clinic measurements, mean (SD)				
BMI (kg/m^2^)	31.3 (6.8)	32.4 (10.6)	30.8 (6.4)	31.6 (8.3)
Total cholesterol (mg/dL)	181.1 (35.4)	182.2 (48.4)	191.6 (35.8)	184.6 (41.0)
LDL-C^d^ (mg/dL)	105.6 (31.8)	103.4 (38.8)	107.0 (28.0)	105.2 (33.5)
HDL-C^e^ (mg/dL)	54.6 (16.9)	53.4 (16.7)	54.8 (15.2)	54.2 (16.2)
Triglycerides (mg/dL)	116.4 (52.5)	107.5 (109.3)	99.9 (52.9)	108.1 (79.6)
Glucose (mg/dL)	109.9 (45.1)	104.5 (32.9)	105.6 (21.0)	106.5 (34.3)
SBP^f^ (mm Hg)	122.7 (17.8)	115.0 (13.8)	126.2 (16.7)	120.8 (16.6)
DBP^g^ (mm Hg)	73.8 (10.0)	81.2 (11.2)	79.5 (8.8)	78.4 (10.6)
HR^h^ (beats/min)	73.0 (14.6)	75.3 (11.0)	67.7 (10.2)	72.3 (12.3)

a BHS: Bogalusa Heart Study.

b NGHS: National Heart, Lung, and Blood Institute Growth and Health Study.

c PLRS: Princeton Lipid Research Study.

d LDL-C:

e HDL-C:high-density lipoprotein cholesterol.

f SBP: systolic blood pressure.

g DBP: diastolic blood pressure.

h HR: heart rate.

The mean age of the participants was 54.2 (SD 8.4) years, which differed somewhat by cohort. Males (13%) were less likely to participate than females (87%), partially due to the NGHS cohort consisting entirely of women. White individuals comprised 69% (93/134) of the participants and Black individuals were 31% (41/134). In-clinic measurements showed a mean BMI in the obese range (31.6, SD 8.3 kg/m^2^), slightly elevated LDL (105.2, SD 33.5 mg/dL), glucose (106.5, SD 34.3 mg/dL), and systolic blood pressure (120.8, SD 16.6 mm Hg), and normal diastolic blood pressure (78.4, SD 10.6 mm Hg), total cholesterol (184.6, SD 41.0 mg/dL), and triglycerides (108.1, SD 79.6 mg/dL).

### Data Collected

**Figure 1.**
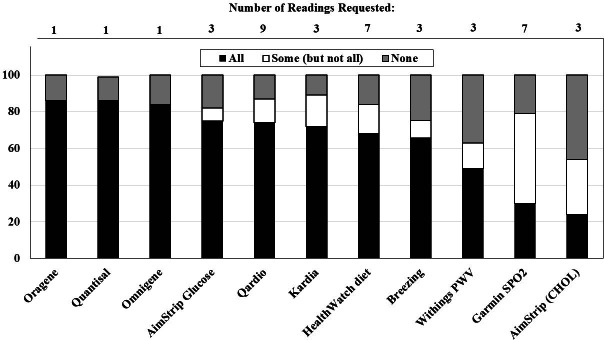
Completion success by device (N=134). PWV: pulse wave velocity, SpO_2_: peripheral capillary oxygen saturation.

Table S1 in Multimedia Appendix 1 lists issues related to device or iPad application set up. General issues encountered included institutional firewalls requiring permission to load apps on an iPad; apps requiring Wi-Fi or a unique email address; complicated instructions for participants; different processes for account setup, data retrieval, cleaning or disinfection by device; need to delete old data or resetting for each device and participant; and differing exported data file formats by device (eg, CSV, PDF requiring data entry, or data entry directly from the device) requiring extensive programming to import into statistical software. Issues encountered during execution of the study (Table S2 in Multimedia Appendix 1) included IRB issues, changes in devices or apps during the study by the manufacturer, issues with batteries, and issues with passwords for apps. Success of completion of the virtual home clinic protocol by device or app type are displayed in Figure 1.

Overall, 122 out of 134 participants (91%) performed at least one of the home tests. Return of biospecimens was highly successful (115/134, 86% returned saliva and 113/134, 84% returned stool). At least 91 of the 134 (68%) participants were able to complete all of the requested readings for glucose, electrocardiogram, BP, and diet record. All requested resting metabolic rate measurements were obtained by 61 of the 93 (66%) NGHS and PLRS participants. The Withings scale that provided weight, body composition, and PWV was more difficult to use but 113 out of 134 (84%) participants provided at least one reading of weight, 109 (81%) provided at least one body composition reading, and 84 (63%) recorded a PWV. Physical activity (steps) and sleep quality and quantity were assessed in 113 out of 134 (84%) participants and oxygen saturation in 106 (79%) participants using the Garmin watch. The AimStrip device to measure total cholesterol resulted in the most calls to the research team and only 32 out of 134 (24%) participants completed all readings. At least one cholesterol result was provided by 72 out of 134 (54%)participants.

Participants were asked whether they preferred home or in-clinic assessment. Of the 95 who responded to this question, 38 (40%) preferred home assessment, 36 (38%) clinic and 21 (22%) did not have a preference. The mean user acceptability score (Table 3) across devices for ease of use was 4.3 (SD 1.0), for instructions was 4.5 (SD 0.7) and for time to use was 3.9 (SD 1.1). The Garmin, Qardio, and Kardia had the highest overall scores (4.7, SD 0.6; 4.6, SD 0.6; and 4.6, SD 0.7) followed by the Withings (4.2, SD 0.9), Breezing and HealthWatch 360 app (4.0, SD 0.7, and 3.9, SD 0.9), with the AimStrip achieving the lowest overall acceptability (3.6, SD 1.2). Greater success with a device was generally not correlated with higher acceptability ratings. This analysis was limited by the fact that participants completing few or no readings were less likely to complete the acceptability survey.

**Table 3.** Mean user acceptability score stratified by device.

Responses (n=100)	Withings	Breezing	Kardia	Qardio	Aim strip	HealthWatch 360	Garmin
Was this device or app easy to use?	4.1 (1.1)	4.1 (0.9)	4.6 (0.9)	4.7 (0.6)	3.6 (1.4)	4.0 (1.1)	4.7 (0.7)
Were the instructions for this device easy to follow?	4.4 (0.8)	4.5 (0.6)	4.8 (0.5)	4.8 (0.5)	4.1 (1.2)	4.5 (0.9)	4.8 (0.6)
How do you feel about the time it took to use the device/app?	3.9 (1.3)	3.4 (1.0)	4.4 (1.1)	4.3 (1.0)	3.2 (1.3)	3.4 (1.3)	4.6 (0.9)
Overall Acceptability Score (mean rating for the 3 factors assessed)	4.2 (0.9)	4.0 (0.7)	4.6 (0.7)	4.6 (0.6)	3.6 (1.2)	3.9 (0.9)	4.7 (0.6)

## Discussion

### Principal Findings

Our study demonstrated that, with careful implementation, collecting at-home biometric measures is feasible and generally acceptable to participants in a longitudinal study. Recently, attention has focused on the use of wearable technology and internet-based applications to capture data that otherwise could only be captured in the clinic. Although some technology, such as accelerometers, have been in use for years [9], there is increasing opportunity to use commercially-available wearable technology and other send-out or mail-back methods to collect epidemiologic data and biospecimens in lieu of clinic visits, even for measurements that have historically been exclusively conducted by medical personnel. However, there remains a considerable gap in knowledge about whether such devices are simple enough to facilitate the collection of valid research-quality data at home and whether these devices are acceptable in terms of ease of use and time commitment for participants participating in mHealth research studies. If these commercially available devices are feasible to use and acceptable to participants, the use of such technologies in extending the reach of epidemiologic studies to widely dispersed populations could revolutionize the field, reduce place-based follow-up bias, and improve the extent of measured data available in follow-up studies.

Our study found 122 out of our 134 (91%) participants performed at least one of the home tests thus demonstrating feasibility. Furthermore, return of biospecimens was highly successful. Home data collection was also highly acceptable with a mean user acceptability score across devices for ease of use of 4.3 out of 5. Interestingly, only 38 out of 95 (40%) participants assessed preferred a home assessment and 36 (38%) preferred in-clinic testing.

There were multiple general implementation issues identified, the majority of which arose because the devices or apps were developed for a single person’s use, while this study required devices to be set up for multiple participants serially over the course of the study. The requirement for a unique email address for many devices, for example, quickly became an issue in this context, as individual participant data needed to be kept separate, but creation of an email address for each participant was not feasible. Device- or app-specific rules about creation or deletion of accounts required detailed protocols for study management. Similarly, data from many of the devices was primarily developed to be viewed in an app or portal by the individual using the device, rather than being exported and used in analyses. Thus, the processes for retrieving the data, and the formats available for the data exports, differed not only across but also within devices, creating complicated data management workflows. While work-arounds were developed to overcome nearly all of the issues, the burden on the study team was higher than for standard clinic measurements.

In terms of the specific devices we tested, our study is the first to date to demonstrate the feasibility and acceptability of the Withings scale to measure body composition in the home. This study also evaluated use of the Breezing in the home setting. All participants repeated the Breezing testing multiple times, and none reported complaints or issues; however, the sample size was small (n=20) and many did occasionally forget to perform the test in the fasting state. The present study did not ask whether participants were fasting for all tests, which would be a modification of study protocol in future studies. Another study evaluated the Breezing in the home in pregnant women [10]. They found 68% of participants performed all the requested readings, although 44% had technical issues [10]. User acceptability was modest with 56% reported they felt the device was enjoyable or had neutral feelings about it, while 44% reported it was not enjoyable [10]. We had higher completion rates and user acceptability but our population was not experiencing any pregnancy-related issues.

There are many studies validating apps to detect arrythmias such as the Kardia [11-14]; however, none of these studies provided user acceptability data. In a study using a different device to detect atrial fibrillation [15], adherence to the study protocol (daily use for a year) was only 44%. Age, severity of symptoms, and BMI influenced adherence but no user acceptability data was provided [15].

There has been a rapid increase in commercially available direct-to-consumer home BP devices of which many, including the Qardio monitor used in this study, have undergone validation studies [16]. No data on acceptability has been published previously. The situation with home oxygen saturation measurements is similar. The Garmin Vivosmart had excellent agreement with a pulse oximeter at rest and 30 seconds after a sit-to-stand and 6-minute walk test [17], but no user acceptability data was provided and the device was not tested in the home. For more complicated hemodynamic measures such as PWV, there are no other commercially available devices for the home besides the Withings [18], and no user-acceptability data has been previously collected.

There are also a variety of commercially available products to measure lifestyle habits such as sleep, physical activity, and diet. The device we used (Garmin Vivosmart 4) has limited published validation data [19], but no studies on user-acceptability data. A study that used a Fitbit for activity monitoring found that 80% of participants provided at least some data during the 3-week study [20]. A few studies have evaluated smartphone apps to track dietary consumption [21], but none have published user acceptability data.

A few studies have examined general participant acceptability of mHealth apps in general. Harmon et al [22] summarized many of the barriers to implementation. For instance, older participants may not have a smartphone or experience in mobile apps. Waning participation over time is also noted when much of the data collection requires active participation from study subjects. Similar to our study, one group found that more than one-third of participants did not wear the device during the study period [23]. Push notifications can assist in reducing this issue but if the device app does not have this feature, sending reminders can be labor intensive. Also, reporting abnormal results requires specific infrastructure and supervision [22]. One study specifically measured acceptability based on a design where a single or multiple devices were used [24]. Although the use of multiple devices gave participants greater interest in the study and more perceived benefit, they had lower confidence in completing the measurements [24]. However, that study was a survey about attitudes toward mHealth studies and did not actually evaluate any specific devices.

Although our sample size is smaller than most epidemiologic studies, it is still larger than many of the mHealth papers that include user acceptability data published to date. We also had a predominance of females, few minorities and the requirement for Wi-Fi access for the participant to complete the at home visit. Although digital literacy was not directly assessed, our sample likely represents a reasonable cross-section of technological proficiency. The most willing and engaged participants likely self-selected themselves for participation as this was a subset of the full cohort. Participants were given some familiarity with the Breezing device during their in-clinic visit, so participants may have felt more comfortable with this device at home than would be the case in a truly remote visit where the participants would only have the study materials provided.

### Conclusion

Our study demonstrated that a virtual home clinic is reasonably feasible and acceptable for remote assessments in longitudinal studies. Challenges for mHealth projects include manufacturer changes to the user interface or data extraction protocols over time, research-specific concerns such as maintaining unique profiles and emails for device apps without requiring the participant to use their personal information, and IRB and institutional concerns about device and participant safety. In addition, over the course of a longer study, the devices themselves might become obsolete and/or no longer supported by the manufacturer. Despite encountered challenges, successful data collection and positive user feedback suggest the potential for revolutionizing epidemiologic studies, especially for widely dispersed populations, and reducing geographic follow-up bias.

## Supplementary material

10.2196/71103 Multimedia Appendix 1 Supplemental tables 1 and 2.

## Abbreviations

BHS: Bogalusa Heart Study
DXA: dual-energy x-ray absorptiometry
I3C: International Childhood Cardiovascular Cohort Consortium
IRB: institutional review board
mHealth: mobile health
MRI: magnetic resonance imaging
NGHS: National Heart, Lung, and Blood Institute Growth and Health Study
NHLBI: National Heart, Lung, and Blood Institute
PLRS: Princeton Lipid Research Study
PWV: pulse wave velocity
