# Supplementary material for: Collecting at-Home Biometric Measures for Longitudinal Research From the i3C: Feasibility and Acceptability Study
Source: JMIR Hum Factors. 2025 Jun 18;12:e71103. doi: 10.2196/71103 (PMC12195623; doi:10.2196/71103)
Supplement: Multimedia Appendix 1 [file humanfactors-v12-e71103-s001.docx]

**Feasibility and Acceptability of Collecting At-Home Biometric Measures for Longitudinal Research**

**Supplemental Tables**

| Supplemental Table 1. Issues encountered with set-up of home devices | | |
| --- | --- | --- |
| Device | Startup Challenges | Startup Solutions |
| Withings Body Cardio Scale | After creating account for a new participant, device no longer synced with iPad via Bluetooth. | Study staff had to select "Forget this Device" in the iPad's Bluetooth settings before re-pairing for a new participant. |
|  | If participant self-reported weight used to set up account was incorrect, scale would not assign measures to correct profile. | Instructions for assigning measures to correct profile given, if necessary. |
|  | App stopped working on older iPads | Updated iPad to newest operating system. |
|  | A valid email address was required to create an account and did not allow deletion of data from an account. | Entire account needed to be deleted entirely between participants, then an iPad-specific email can be used for next person after a waiting period of 7 days. |
|  | Device instructions state Wi-Fi connection needed. | Testing revealed that pairing iPad to device via Bluetooth is sufficient as long as the iPad was connected to Wi-Fi. |
| Breezing Pro™ device | Difficult for participant to use | Additional instruction from CRC provided |
| Alivecor KardiaMobile® 6L | Could not retrieve data because device app required an email account to be set up on the iPad in order to export data, which CCHMC IS does not permit. | Study staff logged into Kardia app on a cell phone using the participant's username/password and email the data from the phone to the study's email address (data being transmitted is deidentified). |
| QardioArm Home BP Monitor | After creating account for a new participant, device no longer synced with iPad via Bluetooth. | Study staff had to select "Forget this Device" in the iPad's Bluetooth settings before re-pairing for a new participant. |
| AimStrip® Tandem | Cord to extract data from device not available. | Study staff retrieved the readings and entered into a REDCap database. |
| GB HealthWatch 360 | Participants may change responses after dietician obtains diet recall | Study staff downloaded data prior to dietician calling participants |
| Garmin Vivosmart® 4 | Initial testing of app on iPad successful but then would not create a new account. | Asking participant to download app to personal phone at in-person visit. Study staff set up account and gave instructions to sync with their phone. Study staff retrieved data from web portal. This issue was resolved a few weeks into the study when the iPad app started working as anticipated again. |
| DNA Genotek™ OMNIgene® GUT OMR-200 stool collection | Required special packaging for exempt human specimen | Instructions for use of compliant packaging provided |
| DNA Genotek™ Oragene® OG-500 & Abbott Quantisal™ saliva collection | Inadequate sample size may be obtained | Study staff performed web-based instruction and sent another kit. |

| Supplemental table 2. Issues with execution of the study. | | |
| --- | --- | --- |
| Device | Challenges after Startup | Solutions after Startup |
| Withings Body Cardio Scale | Withings changed the look of their iPad app icon several times during the study. | IRB modifications required in order to update the icon image in the participant-facing instructions. |
|  | Some participants were unable to get the five successful readings in a row required to obtain a PWV measurement. | Remote troubleshooting attempted by study staff where possible. |
|  | After deleting account, the associated email address was sometimes not able to be reused after the 7-day waiting period for unknown reasons. | No solution found. |
|  | Layout and functioning of app changed multiple times over the course of the study. | Manual of procedures for device setup and data retrieval updated as needed. |
|  |  |  |
| Breezing Pro™ device | Chip slot's spring mechanism broke during participant use on two of the devices. | Manufacturer replaced first broken device. Did not request replacement for second. |
| Alivecor KardiaMobile®  6L | Tulane site unable to view ECG history on cell phone app in order to export the pdf files of the results and tracings. | Study staff from Cincinnati downloaded and emailed the files to Tulane. |
| QardioArm Home BP Monitor | App does not seem to indicate when batteries are running low. Batteries died during a few participants' virtual clinic weeks. | Study staff replaced batteries pre-emptively in all other Qardio devices. |
|  | Several of our larger patients were unable to successfully and/or comfortably use the monitor due to cuff size. | No solution found. |
| AimStrip® Tandem | Many participants unable to successfully complete a cholesterol reading, receiving an error message on the meter. | No solution found. It appeared that the meter required a larger amount of blood on the cholesterol test strip than most participants were able to obtain from a finger stick. |
|  | Devices and instructions sometimes returned with dried blood on them. | Meter, lancing device, and instructions had to be *very* carefully checked and cleaned between participants. Soiled instruction sheets were replaced. Though thorough disinfection of the device between participants was always planned, checking the instruction sheets for blood had to be added to the procedure. |
| GB HealthWatch 360 | Feedback from a number of participants indicated they had trouble figuring out how to enter many of their foods, especially those cooked from scratch at home. | Participants advised to select the closest approximation of what they ate even if not an exact match. |
|  | Several participants got logged out of the app unexpectedly but did not follow the provided instructions re: how to log back in and instead created a new account using their personal email address. | No solution found. Unable to run reports for these participants on the GB HealthWatch website since the account they created was not linked to our study portal. |
| Garmin Vivosmart® 4 | With Pulse Ox measurement enabled, watch battery drained very quickly. | If a participant's device box needed to be prepared a few days in advance, Garmin had to be removed from box the morning of the clinic visit to be recharged. |
|  | Even when pulse ox measurement is enabled, watch does not successfully collect SPO2 data every night. For some nights participants wore the watch, we were able to see sleep stage data but not SPO2. | No solution found. Unclear what caused the issue. |
|  | A sleep data report had been available was eliminated from the Garmin web portal mid-way through the study. The way pulse ox data was displayed was also changed. | Though the sleep data report was not available for participants enrolled after a certain date, the same data was able to be entered into REDCap from the website dashboard. Manual of procedures for retrieving data was updated based on the new pulse ox display. |
| iPad | Passcode to open iPad sometimes expired while a participant had it at home, prompting them to set a new passcode. | One participant changed the passcode but wrote down the new one for us so that we were still able to log in. Others have contacted us to find out what they should do. Passcodes can be preemptively changed before a year has passed to avoid this (we were originally unaware they would expire). |
| General | Several participants have returned kits smelling strongly of cigarette smoke. | When possible, we have let these kits "air out" for a while before reusing. Even with an airing out period and thorough cleaning, we were not able to eliminate the odor entirely. |
